# Supplementary material for: Recurring marine phosphorus spikes during major palaeozoic mass extinctions and climate change
Source: Nat Commun. 2026 Mar 24;17:4481. doi: 10.1038/s41467-026-70701-y (PMC13187173; doi:10.1038/s41467-026-70701-y)
Supplement: Supplementary file 1 — Supplementary Information [file 41467_2026_70701_MOESM1_ESM.pdf]

Supplementary Notes for

Recurring Marine Phosphorus Spikes during Major Palaeozoic Mass Extinctions and Climate Change

Matthew S. Dodd[1,2,3]*, Chao Li[2,3]*, Zihu Zhang[2,3], Aleksey Y. Sadekov[1], André Desrochers[4,5], Olle Hints[6], Detian Yan[7], Xiangrong Yang[7,8], Annette D. George[1], Maya Elrick[9], David White[9], Wenkun Qie[10], Bo Chen[10], Andrew S. Merdith[11], Benjamin J. W. Mills[12]

[1] School of Earth and Oceans, University of Western Australia, Perth, WA 6009, Australia
[2] State Key Laboratory of Oil and Gas Reservoir Geology and Exploitation & Institute of Sedimentary Geology,
Chengdu University of Technology, Chengdu 610059, China
[3] International Center for Sedimentary Geochemistry and Biogeochemistry Research, Chengdu University of Technology, Chengdu 610059, China
[4] Earth and Environmental Sciences, University of Ottawa, Ottawa, Ontario, Canada, K1N 6N5
[5]Société du patrimoine mondial Anticosti, Port-Menier, Québec, Canada, G0G 2Y0
[6] Department of Geology, Tallinn University of Technology, Ehitajate tee 5, 19086 Tallinn, Estonia
[7] Key Laboratory of Tectonics and Petroleum Resources of Ministry of Education, China University of Geosciences, Wuhan 430074, China
[8] School of Geosciences, Yangtze University, Wuhan 430100, China
[9] Earth and Planetary Sciences, University of New Mexico, Albuquerque, NM 87131, United States of America
[10] State Key Laboratory of Palaeobiology and Stratigraphy, Nanjing Institute of Geology and Palaeontology, Chinese Academy of Sciences, Nanjing 210008, China
[11] School of Physics, Chemistry and Earth Sciences, The University of Adelaide, Adelaide, 5005, SA, Australia
[12] School of Earth and Environment, University of Leeds, Leeds, LS2 9JT, UK
*These authors contributed equally
matthew.dodd@uwa.edu.au; chaoli@cdut.edu.cn

Supplementary Notes contents:

Supplementary Note 1: Study regions and samples

Supplementary Note 2: An in-situ Laser ablation study of CAP heterogeneity and preservation in bioclastic limestone

Supplementary Note 3: Controls on the incorporation of CAP into carbonate

Supplementary Note 1: Study regions and samples

Geological sections for the Late Ordovician Mass Extinction Event

Anticosti, Canada

Anticosti Island exhibits an expanded, largely undeformed sedimentary succession that dips gently to the southwest, spanning the Ordovician-Silurian boundary [1,2]. During the Late Ordovician, the Anticosti Basin was positioned along the southeastern margin of Laurentia, between 20° and 30°S paleolatitude [3,4]. The facies predominantly reflect storm-dominated deposition in shallow marine settings, consistent with the basin's location within the tropical storm belt [2,5].

The Hirnantian Stage on Anticosti Island encompasses the entire Ellis Bay Formation, although some authors restrict it to the formation's uppermost portion [6-8]. The broader interpretation is supported by multiple sedimentological and stratigraphic lines of evidence [9-13]. Previous studies have identified a hierarchical stacking of sedimentary cycles characterized by alternating deepening and shallowing trends, attributed to the waxing and waning of continental ice sheets over Gondwana [1,12,14].

The basal contact of the biohermal/oncolitic limestones near the top of the Ellis Bay Formation (i.e. the base of the Laframboise Member) is a regionally traceable sequence boundary marked by abrupt facies shifts and erosion, interpreted as recording peak late Hirnantian regression and erosion [1,12,14]. The biohermal/oncolitic limestones were deposited during the subsequent post-glacial transgression. The Ellis Bay-Becscie contact (i.e. the top of the Laframboise Member) marks a second stratigraphic surface, locally associated with erosion and hardground development, and may represent a transgressive ravinement surface formed during the more rapid post-glacial sea-level rise into the latest Hirnantian and early Silurian. This stratigraphic interval encompasses the second extinction pulse of the Late Ordovician Mass Extinction, LOME-2 [1,12,14].

Viki drill core, Estonia

The Baltica palaeocontinent was located between the equator and 30°S latitudes during the latest Ordovician [15]. Ordovician-Silurian carbonate rocks are widespread and well-preserved in the Baltic region, notably in Estonia, representing deposits of the epicontinental Baltic paleobasin [16,17].

The Viki reference drill core comes from Saaremaa Island, Estonia (58.35107°N, 22.07985°E) and uncovers a Middle Ordovician to Ludlow carbonate sequence [18]. It has been thoroughly sampled for microfossils and geochemistry and referred to in numerous publications [19-21].

The Viki locality represents transitional area in the Late Ordovician basin between the marginal shallow shelf settings in the north (Estonian Shelf *sensu* [22] and deeper-water environments in the south (Livonian Basin). The Hirnantian rocks are shallow-water carbonates (skeletal and oolitic grainstone, argillaceous and dolomitised partly laminated limestone and carbonate mudstone) assigned to the Ärina and Saldus formations by Põldvere (2010). Carbon isotope chemostratigraphy suggests that the lowermost part of the Varbola Formation is most likely also of Hirnantian age, with the HICE identified at core depths of ca 240–246 m [20,23]. The occurrence of conodont *Noixodontus girardeauensis* further constrains the Saldus Formation to the Hirnantian [24]; however, graptolites are missing and precise correlation with graptolite zones remains therefore tentative. Abrupt changes in lithology as well as chemostratigraphy suggest the presence of gaps in the succession, related to the Hirnantian sea level changes.

Wukemuchang, South China

The South China Block was located near the equator during the Late Ordovician (Fig. 2), and it comprised the Cathaysia Block in the southeast and the Yangtze

Block in the northwest [25,26]. The northern part of the Yangtze Block formed a deep-water depressional basin as a result of the collision between the Yangtze and Cathaysia Blocks [25,26].

The Wukemuchang (Wuke) section is a shallow-shelf carbonate section, which is located in the southwestern part of Yangtze Block. This section can be divided into the Baota/Linxiang Formation, Tiezufeike Formation, and Butuo Formation, spanning the Late Ordovician to the Early Silurian [27-29]. The Baota and Linxiang formations are mainly characterized by limestone. The Tiezufeike Formation mainly comprises limestone and dolomitic limestone, with the upper Tiezufeike Formation containing abundant shelly fauna (i.e. the Hirnantian fauna). The Butuo Formation comprises laminated limestone and calcareous mudstones interbedded with argillaceous siltstone and mudstone.

Geological sections for the Late Devonian Mass Extinction Event

Horse Spring (HS) drill core, Western Australia

A 323-meter-thick continuous drill core from the Horse Spring Range intersects a toe-of-slope to lower–middle fore-reef slope succession spanning the Frasnian–Famennian boundary [30]. The core consists of unweathered material, making it ideal for geochemical analysis and conodont biostratigraphy to locate the boundary and place it within the regional sea-level history [31,32].

The basal facies are gray laminated siltstones of the basinal Gogo Formation overlain by facies of the Virgin Hills Formation represented by a coarsening-upward succession of red siltstones, calcareous siltstones, and nodular to very thinly bedded limestones. These are overlain by red, turbiditic packstones–grainstones and

breccias. A distinctive 3.2-meter-thick interval in the lower part of the core contains three discrete olive-green–gray siltstone layers (0.25–0.3 m thick), each with a 2–3 cm dark gray siltstone layer. A 0.1-meter-thick micaceous sandstone layer is present at 263 meters. Nodular to bedded limestones throughout the core contain scattered crinoidal and shelly debris, with small fragments of *Amphipora* stromatoporoids present in the lower ~40 meters. Reefal limestone (*Renalcis*-bearing microbialite) is common in the Famennian breccias and as meter-scale blocks, reflecting a stratigraphic distribution similar to other fore-reef slope measured sections along the northern margin of the Canning Basin [33,34]. The drillcore succession closely resembles the nearby Horse Spring section, where detailed conodont biostratigraphy was previously used to define the Frasnian–Famennian boundary [35,36].

Conodont biostratigraphy indicates depositional ages ranging from Frasnian Zone MN 6 at the base of the succession to the Famennian *marginifera* Zone at the top. The base of the Famennian is typically defined by the first occurrence of *Palmatolepis subperlobata* within the *triangularis* Zone (Klapper, 2007), however, *Pa. subperlobata* is absent from the HS core. Hence, the position of the Frasnian–Famennian boundary lies between the lowest occurrence of *Palmatolepis* taxa indicative of the *triangularis* Zone (notably *Pa. triangularis*) at 215.6 meters and the youngest age-diagnostic Frasnian sample (conodont Zone MN 13) at 218.6 meters (George et al. 2014).

Devils Gate, Nevada

During the Late Devonian, Laurentia straddled the paleoequator and was separated from Gondwana by the narrow and closing Rheic ocean (Fig. 2). Globally high sea

levels resulted in extensive continental flooding and the development of vast epeiric seas.

Along western Laurentia, the westward-deepening continental margin accumulated nearly 8 km of Proterozoic through Upper Devonian siliciclastic and carbonate passive-margin deposits. By the Late Devonian, convergence related to the Antler orogeny along western Laurentia resulted in the development of a foreland basin and forebulge atop the passive-margin succession [37].

At the Devil's Gate section of central Nevada, the deposition related to the transition between passive- and convergent-margin tectonics includes the Upper Devonian upper member of the Devil's Gate Limestone, which accumulated in middle through outer ramp and ramp-slope environments. At this location, the Devil's Gate Limestone has excellent age control using detailed conodont biostratigraphy [38] and previously established $\delta^{13}C$ trends [39]. For this study, we identified six depositional facies in the ~140 m-thick studied succession, and these facies stack into six My-scale depositional sequences. Three of these sequences are correlated across Laurentia and into Europe, indicating they were driven by global sea-level changes [40].

Yangdi, South China

During the Late Devonian, South China was positioned near the equator along the eastern margin of the Paleotethys. From the late Emsian to the Famennian, a network of NE- and NW-trending synsedimentary faults cut through the basement of the region's epicontinental sea, resulting in a complex facies architecture. These intersecting fault blocks led to the development of shallow-water carbonate platforms separated by deep-water basins. This depositional differentiation is well-documented

in local geological literature as the Xiangzhou-type shallow-water facies and the Nandan-type deep-water facies [41,42].

The Yangdi section (24° 58.2′ N, 110° 22.8′ E), also referred to as Fuhe in other studies (e.g.,[42]), is exposed along a roadcut between Baoan and Yangdi County, approximately 35 km southeast of Guilin City, Guangxi Province, South China. During the Late Devonian, this site was part of a spindle- to rhombic-shaped transtensional intraplatform basin, encircled by shallow-water carbonate platforms [42-44].

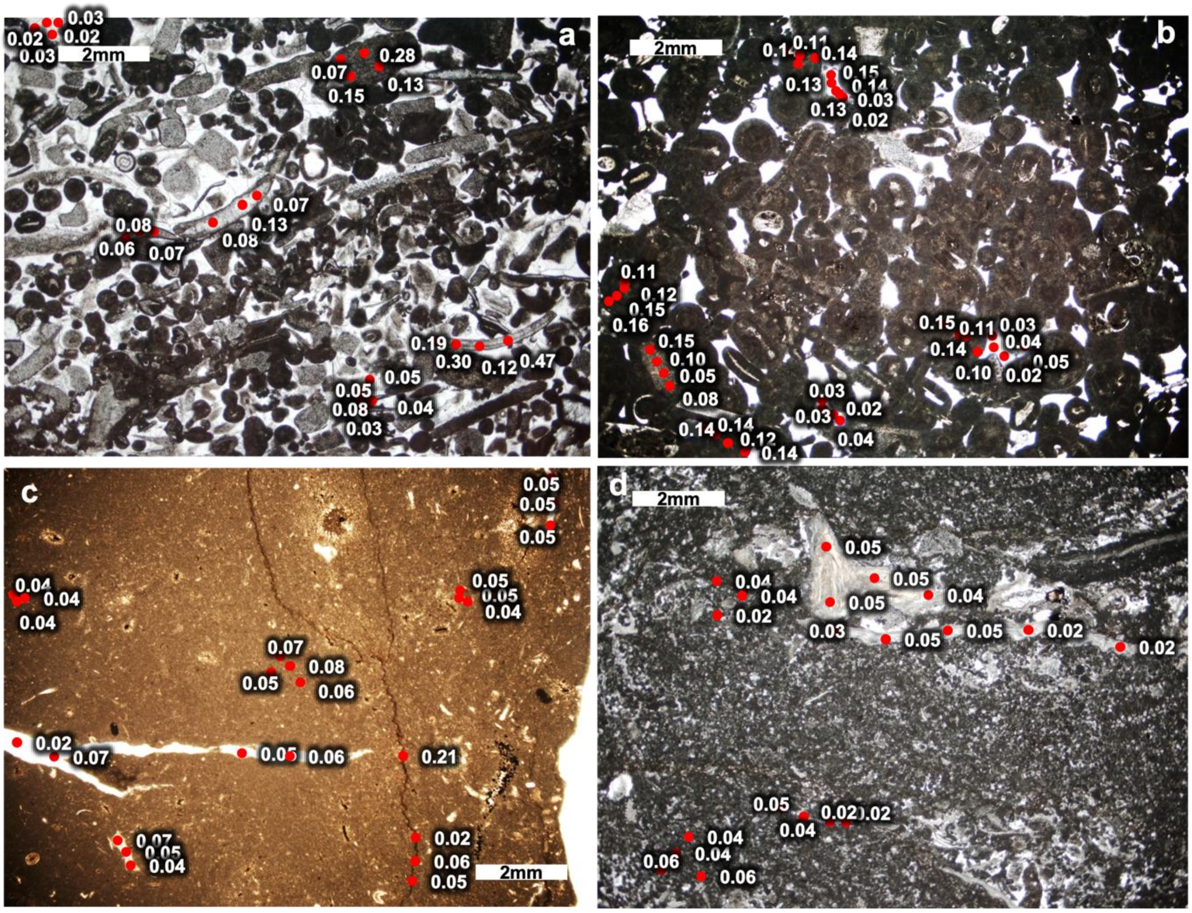

Supplementary Figure 1. Photomicrographs of samples (a) 244.95, (b) 244.55, (c) 242.75, (d) 245.14 and laser ablation targets with CAP values from the Viki drill

core. All CAP values displayed are in mmol/ mol. Red dots correspond to laser ablation spots with adjacent CAP value.

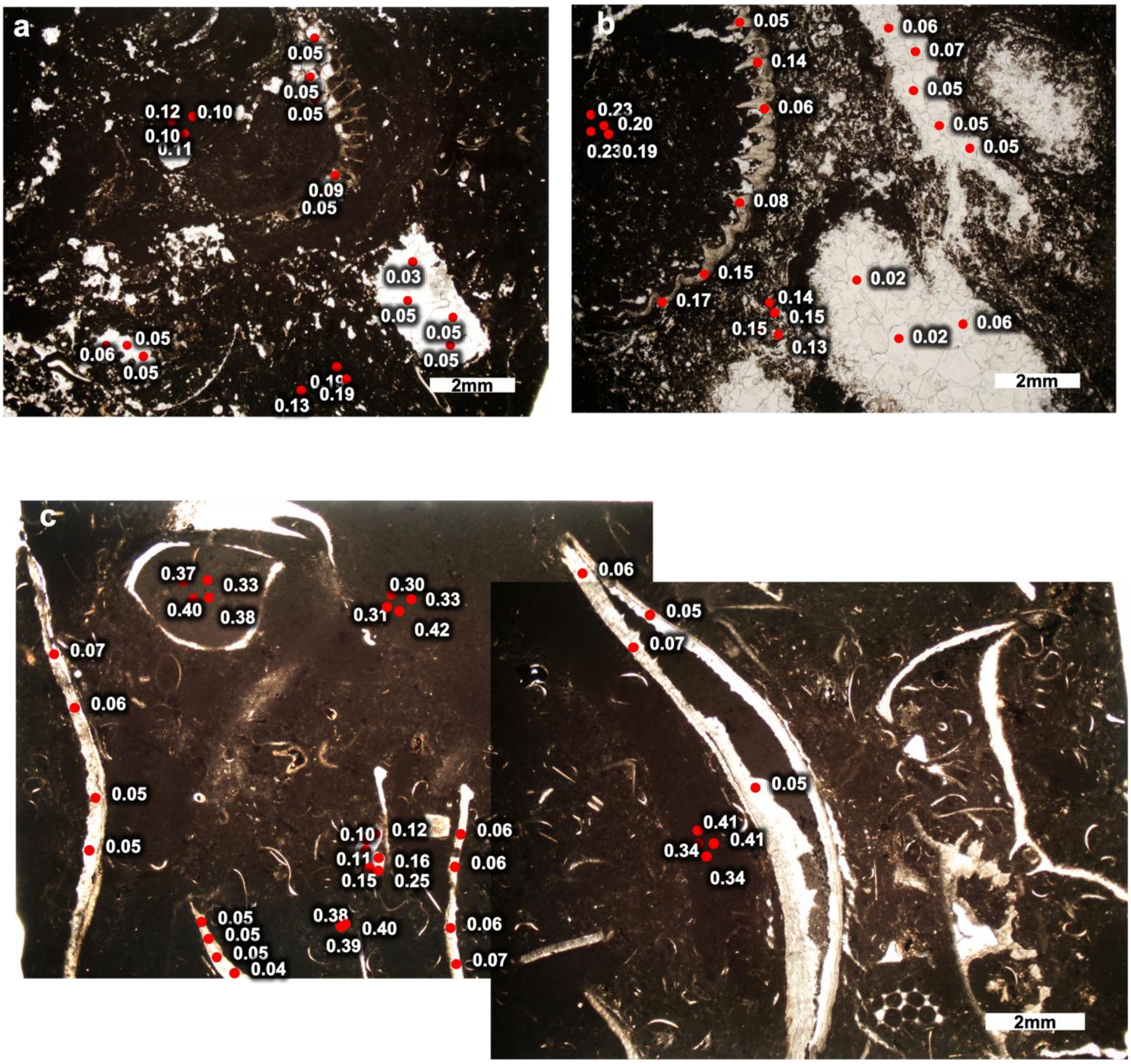

Supplementary Figure 2. Photomicrographs of samples (a) A24, (b) A60, (c) A45 and laser ablation targets with CAP values from Pointe Laframboise and Ellis Bay sections on Anticosti Island, Canada. Red dots correspond to laser ablation spots with adjacent CAP value.

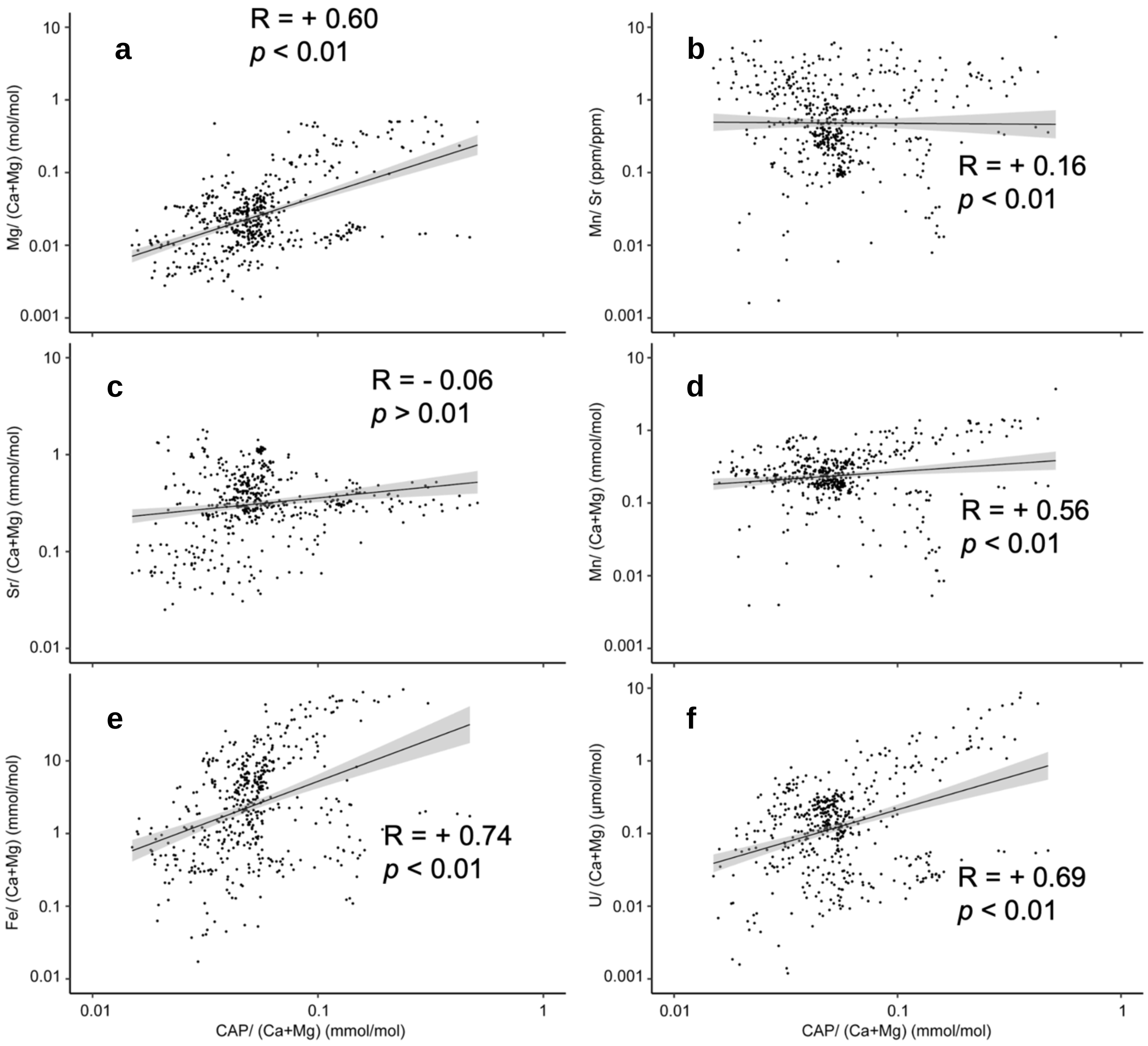

Supplementary Figure 3. Cross plots of carbonate trace element data from laser ablation points in Ordovician samples from the Viki core. a) Mg/ (Ca+Mg) vs. CAP/ (Ca+Mg); b) Mn/ Sr vs. CAP/ (Ca+Mg); c) Sr/ (Ca+Mg) vs. CAP/ (Ca+Mg); d) Mn/ (Ca+Mg) vs. CAP/ (Ca+Mg); e) Fe/ (Ca+Mg) vs. CAP/ (Ca+Mg); f) U/ (Ca+Mg) vs. CAP/ (Ca+Mg).

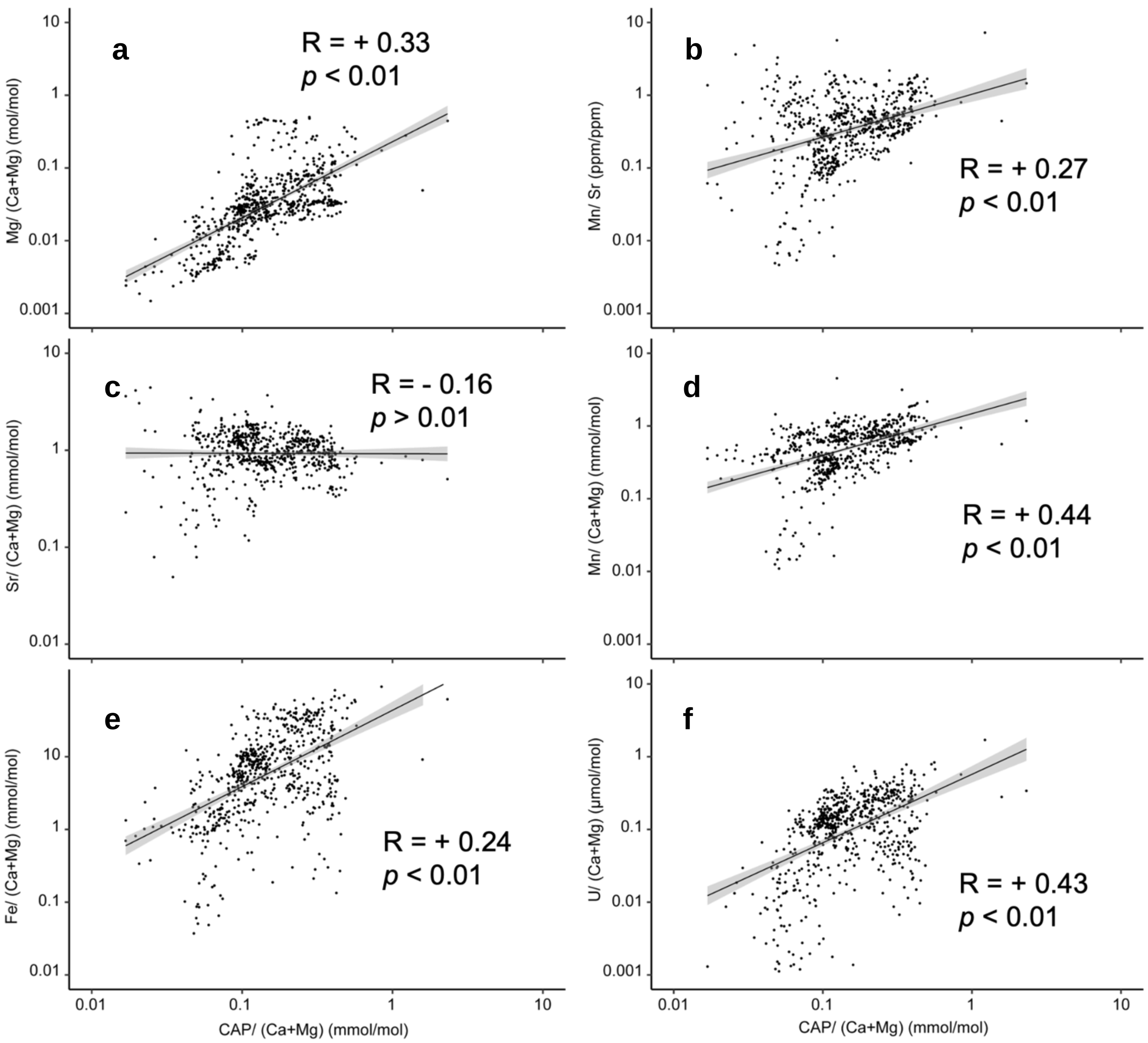

Supplementary Figure 4. Cross plots of carbonate trace element data from laser ablation points in Ordovician samples from Anticosti island. a) Mg/ (Ca+Mg) vs. CAP/ (Ca+Mg); b) Mn/ sr vs. CAP/ (Ca+Mg); c) Sr/ (Ca+Mg) vs. CAP/ (Ca+Mg); d) Mn/ (Ca+Mg) vs. CAP/ (Ca+Mg); e) Fe/ (Ca+Mg) vs. CAP/ (Ca+Mg); f) U/ (Ca+Mg) vs. CAP/ (Ca+Mg).

Supplementary Table 1. Bulk rock geochemical data statistics for key indicators of diagenetic alteration. Emboldened numbers represent instances where a statistically significant (<0.01) correlations occur.

Section	Statistical measure	$\delta^{13}C_{carb}$ vs. CAP	$\delta^{18}O_{carb}$ vs. CAP	Mg/ Ca vs. CAP	Fe vs. CAP	Sr vs. CAP	La vs. CAP	U vs. CAP	Mn/ Sr vs. CAP
Pointe Laframboise	R	0.04	0.46	-0.33	-0.36	0.28	0.42	0.20	0.09
	p	>0.01	>0.01	>0.01	>0.01	>0.01	>0.01	>0.01	>0.01
Ellis Bay	R	-0.28	-0.12	0.13	0.03	-0.36	0.45	0.25	0.43
	p	>0.01	>0.01	>0.01	>0.01	>0.01	>0.01	>0.01	>0.01
Viki core	R	0.58	0.31	0.01	0.02	-0.24	-0.54	-0.33	0.31
	p	**<0.01**	>0.01	>0.01	>0.01	>0.01	**<0.01**	>0.01	>0.01
Wukemuchang	R	-0.53	0.74	-0.03	-0.29	-0.19	0.02	0.24	-0.12
	p	**<0.01**	>0.01	>0.01	>0.01	>0.01	>0.01	>0.01	>0.01
HS core	R	-	-	0.09	-0.16	-0.01	-0.04	-0.21	0.06
	p	>0.01	>0.01	>0.01	>0.01	>0.01	>0.01	>0.01	>0.01
Yangdi	R	-	-	0.05	0.21	0.03	0.09	-	-0.04
	p	>0.01	>0.01	>0.01	>0.01	>0.01	>0.01	>0.01	>0.01
Devil's Gate	R	-0.25	0.1	0.01	-0.04	0.5	0.14	0.02	-0.39
	p	>0.01	>0.01	>0.01	>0.01	**<0.01**	>0.01	>0.01	>0.01

Supplementary Note 2: An in-situ Laser ablation study of CAP heterogeneity and preservation in bioclastic limestone

Given the complex mixtures of biological and abiotic carbonate phases in marine carbonate sediments, we conducted a detailed study of CAP in various carbonate phases from select samples across our study sections in the Ordovician Anticosti and Viki sections, in order to compare with our bulk rock CAP extracts. In-situ laser ablation analyses have provided evidence for late-stage diagenetic alteration of carbonate in the Viki drill core (Hagen-Peter et al., 2021). Interstitial calcite cement displays various characteristics, of diagenetic calcite, in particular low Sr and elevated Mn/Sr, whereas bioclastic grains retain more primary marine-carbonate trace element characteristics (e.g., high Sr). Along with elevated U concentrations, which has been taken as an indicator for well-preserved carbonate chemistry [45,46]. Bioclastic limestones provided U-Pb ages that are consistent with the depositional age, while the interstitial calcite spar gave ages that were tens of millions of years younger, which support their post-depositional origins [19]. Correlations of CAP, Mg and U may be expected given that these trace elements will often be removed from the carbonate lattice as amorphous precursors transition into well-crystallised forms [47]. Consequently, the higher CAP values in the bioclastic fractions likely represent the closest values to the original primary signature. Conversely, in the Anticosti samples, the calcite matrix has CAP values that are higher than those of the bioclasts (Supplementary Information Figure 2). These differences likely reflect the depositional history of the carbonate rock, with the Viki samples mostly being grain supported, with the interstitial calcite being a late diagenetic phase, whereas the matrix supported calcite in the Anticosti samples, likely reflects primary micrite precipitates. The purpose of the *in-situ* analysis was to identify diagenetic trends in CAP, not to produce a more reliable CAP record. It may be possible to selectively

target only the most “pristine” carbonate phases based on petrographic characteristics using laser ablation; however, diagenetically altered carbonates are not always identifiable petrographically, and that is why we focus on using the CAP and trace element data trends rather than data points picked from petrographically chosen targets.

While CAP trends from bulk rock and *in-situ* analyses are consistent, their absolute values differ. This may arise from incomplete recovery of P during bulk rock extractions, combined instrument- and method-specific errors, and sampling bias during target acquisition for *in-situ* laser ablation. Sampling bias was likely the biggest factor, as analyses were focused on obtaining data points from a variety of mineral phases as opposed to a homogenous sampling approach.

Supplementary Note 3: Controls on the incorporation of CAP into carbonate

Aside from phosphate concentration in seawater, other factors such as seawater pH, alkalinity, temperature and precipitation rate can affect CAP uptake in carbonate [48,49], therefore the observed trends in CAP could reflect a change in seawater chemistry, as opposed to an increase in phosphate concentration. It would be intuitive that an expanded glacial period would impact ocean chemistry. For example, lithium isotope data during the Hirnantian interval imply significant changes in silicate weathering and by extension atmospheric CO_2 levels, which in turn can affect ocean pH, alkalinity, temperature and carbonate saturation states. A positive lithium isotope excursion immediately following the CAP spike has been taken as evidence for a decline in atmospheric CO_2 [50], therefore the decline in CAP values following the CAP spike may reflect an increase in ocean pH or alkalinity or both (Fig. 1). However, a return to pre-excursion δ^7Li_{carb} values and presumably roughly

equivalent weathering rates and CO_2 levels does not result in a rebound in CAP values. Therefore, it appears unlikely that CAP trends were significantly impacted by changing CO_2 levels and the resulting impact on ocean chemistry. Similarly, CAP and oxygen isotope data from conodont and carbonate (proxy for ocean temperature) do not correlate in the Hirnantian age Anticosti samples, and in the Viki drill core peak CAP values coincide with peak $\delta^{18}O_{carb}$ values and therefore minimum ocean temperatures (and likely by extension lower atmospheric CO_2) (Fig. 1). This provides confidence that the CAP trends are unlikely to result from changing oceanic temperature, or a decrease in ocean pH, alkalinity or precipitation rate that would result from shifting atmospheric CO_2 levels. Consequently, the CAP trends can be convincingly considered to principally record changes in seawater P concentration.

References:

1 Desrochers, A., Farley, C., Achab, A., Asselin, E. & Riva, J. F. A far-field record of the end Ordovician glaciation: The Ellis Bay Formation, Anticosti Island, Eastern Canada. *Palaeogeography, Palaeoclimatology, Palaeoecology* **296**, 248-263 (2010). https://doi.org/https://doi.org/10.1016/j.palaeo.2010.02.017

2 Long, D. G. F. Tempestite frequency curves: a key to Late Ordovician and Early Silurian subsidence, sea-level change, and orbital forcing in the Anticosti foreland basin, Quebec, Canada. *Canadian Journal of Earth Sciences* **44**, 413-431 (2007). https://doi.org/10.1139/e06-099

3 Jin, J. *et al.* Precisely locating the Ordovician equator in Laurentia. *Geology* **41**, 107-110 (2013). https://doi.org/10.1130/G33688.1

4 Scotese Christopher, R. Ordovician plate tectonic and palaeogeographical maps. *Geological Society, London, Special Publications* **532**, 91-109 (2023). https://doi.org/10.1144/SP532-2022-311

5 Sami, T. & Desrochers, A. Episodic sedimentation on an early Silurian, storm-dominated carbonate ramp, Becscie and Merrimack formations, Anticosti Island, Canada. *Sedimentology* **39**, 355-381 (1992). https://doi.org/https://doi.org/10.1111/j.1365-3091.1992.tb02122.x

6 Bergström, S. M., Young, S. & Schmitz, B. Katian (Upper Ordovician) δ13C chemostratigraphy and sequence stratigraphy in the United States and Baltoscandia: A regional comparison. *Palaeogeography, Palaeoclimatology, Palaeoecology* **296**, 217-234 (2010). https://doi.org/https://doi.org/10.1016/j.palaeo.2010.02.035

7 Brenchley, P. J. *et al.* High-resolution stable isotope stratigraphy of Upper Ordovician sequences: Constraints on the timing of bioevents and

environmental changes associated with mass extinction and glaciation. *GSA Bulletin* **115**, 89-104 (2003). https://doi.org/10.1130/0016-7606(2003)115<0089:HRSISO>2.0.CO;2
8 Brenchley, P. J. *et al.* Bathymetric and isotopic evidence for a short-lived Late Ordovician glaciation in a greenhouse period. *Geology* **22**, 295-298 (1994). https://doi.org/10.1130/0091-7613(1994)022<0295:BAIEFA>2.3.CO;2
9 Achab, A., Asselin, E., Desrochers, A., Riva, J. F. & Farley, C. Chitinozoan biostratigraphy of a new Upper Ordovician stratigraphic framework for Anticosti Island, Canada. *GSA Bulletin* **123**, 186-205 (2011). https://doi.org/10.1130/B30131.1
10 Mauviel, A. & Desrochers, A. A high-resolution, continuous δ13C record spanning the Ordovician–Silurian boundary on Anticosti Island, eastern Canada. *Canadian Journal of Earth Sciences* **53**, 795-801 (2016). https://doi.org/10.1139/cjes-2016-0003
11 Mauviel, A., Sinnesael, M. & Desrochers, A. The stratigraphic and geochemical imprints of Late Ordovician glaciation on far-field neritic carbonates, Anticosti Island, eastern Canada. *Palaeogeography, Palaeoclimatology, Palaeoecology* **543**, 109579 (2020). https://doi.org/https://doi.org/10.1016/j.palaeo.2019.109579
12 Zimmt, J. B., Holland, S. M., Desrochers, A., Jones, D. S. & Finnegan, S. A high-resolution sequence stratigraphic framework for the eastern Ellis Bay Formation, Canada: A record of Hirnantian sea-level change. *GSA Bulletin* **136**, 3825-3849 (2024). https://doi.org/10.1130/B37190.1
13 Zimmt, J. B. & Jin, J. A new species of Hirnantia (Orthida, Brachiopoda) and its implications for the Hirnantian age of the Ellis Bay Formation, Anticosti Island, eastern Canada. *Journal of Paleontology* **97**, 47-62 (2023). https://doi.org/10.1017/jpa.2022.83
14 Ghienne, J.-F. *et al.* A Cenozoic-style scenario for the end-Ordovician glaciation. *Nature Communications* **5**, 4485 (2014). https://doi.org/10.1038/ncomms5485
15 Cocks, L. R. M. & Torsvik, T. H. Ordovician palaeogeography and climate change. *Gondwana Research* **100**, 53-72 (2021). https://doi.org/https://doi.org/10.1016/j.gr.2020.09.008
16 Nestor, H. & Einasto, R. in *Geology and Mineral Resources of Estonia* (eds A. Raukas & A. Teedumäe) 192-204 (Estonian Academy Publishers, 1997).
17 Meidla, T., Ainsaar, L., Hints, O. & Radzevičius, S. Ordovician of the Eastern Baltic palaeobasin and the Tornquist Sea margin of Baltica. *Geological Society, London, Special Publications* **532**, 317-343 (2023). https://doi.org/10.1144/SP532-2022-141
18 Põldvere, A. in *Estonian Geological Sections Bulletin 10* 56 (Geological Survey of Estonia, 2010).
19 Hagen-Peter, G., Wang, Y., Hints, O., Prave, A. R. & Lepland, A. Late diagenetic evolution of Ordovician limestones in the Baltoscandian basin revealed through trace-element mapping and in situ U–Pb dating of calcite. *Chemical Geology* **585**, 120563 (2021). https://doi.org/https://doi.org/10.1016/j.chemgeo.2021.120563
20 Hints, O. *et al.* Paired carbon isotope chemostratigraphy across the Ordovician–Silurian boundary in central East Baltic: Regional and global signatures.

Palaeogeography, Palaeoclimatology, Palaeoecology **624**, 111640 (2023). https://doi.org/https://doi.org/10.1016/j.palaeo.2023.111640
21 Thiagarajan, N. *et al.* Reconstruction of Phanerozoic climate using carbonate clumped isotopes and implications for the oxygen isotopic composition of seawater. *Proceedings of the National Academy of Sciences* **121**, e2400434121 (2024). https://doi.org/10.1073/pnas.2400434121
22 Harris, M. T. *et al.* Upper Ordovician sequences of western Estonia. *Palaeogeography, Palaeoclimatology, Palaeoecology* **210**, 135-148 (2004). https://doi.org/https://doi.org/10.1016/j.palaeo.2004.02.045
23 Hints, O. *et al.* New data on Ordovician stable isotope record and conodont biostratigraphy from the Viki reference drill core, Saaremaa Island, western Estonia. *GFF* **136**, 100-104 (2014). https://doi.org/10.1080/11035897.2013.873989
24 Männik, P. in *Viki Drill Core* (ed A Põldvere) 21-24 (Geological Survey of Estonia, 2010).
25 Xu, C., Jia-yu, R., Yue, L. & Boucot, A. J. Facies patterns and geography of the Yangtze region, South China, through the Ordovician and Silurian transition. *Palaeogeography, Palaeoclimatology, Palaeoecology* **204**, 353-372 (2004). https://doi.org/https://doi.org/10.1016/S0031-0182(03)00736-3
26 Xu, C., Yuandong, Z., Junxuan, F., Lan, T. & Haiqing, S. Onset of the Kwangsian Orogeny as evidenced by biofacies and lithofacies. *Science China-earth Sciences* **55**, 1592-1600 (2012).
27 Liu, M. *et al.* Oceanic anoxia and extinction in the latest Ordovician. *Earth and Planetary Science Letters* **588**, 117553 (2022). https://doi.org/https://doi.org/10.1016/j.epsl.2022.117553
28 Yang, X. *et al.* Spatiotemporal variations of sedimentary carbon and nitrogen isotopic compositions in the Yangtze Shelf Sea across the Ordovician-Silurian boundary. *Palaeogeography, Palaeoclimatology, Palaeoecology* **567**, 110257 (2021). https://doi.org/https://doi.org/10.1016/j.palaeo.2021.110257
29 Yang, X. *et al.* Lithium isotope and mercury evidence for enhanced continental weathering and intense volcanism during the Ordovician-Silurian transition. *Geochimica et Cosmochimica Acta* **391**, 49-68 (2025). https://doi.org/https://doi.org/10.1016/j.gca.2024.12.010
30 George, A. D., Chow, N. & Trinajstic, K. M. Oxic facies and the Late Devonian mass extinction, Canning Basin, Australia. *Geology* **42**, 327-330 (2014). https://doi.org/10.1130/G35249.1
31 George, A., D., Trinajstic, K., M. & Chow, N. Frasnian reef evolution and palaeogeography, SE Lennard Shelf, Canning Basin, Australia. *Geological Society, London, Special Publications* **314**, 73-107 (2009). https://doi.org/10.1144/SP314.4
32 George Annette, D., Seyedmehdi, Z. & Chow, N. in *West Australian Basin Symposium 2013* (Petroleum Exploration Society of Auatralia 2013).
33 Chow, N., George, A. D. & Trinajstic, K. M. Tectonic control on development of a Frasnian-Famennian (Late Devonian) palaeokarst surface, Canning Basin reef complexes, northwestern Australia. *Australian Journal of Earth Sciences* **51**, 911-917 (2004). https://doi.org/10.1111/j.1400-0952.2004.01093.x

34 George, A. D. & Chow, N. The depositional record of the Frasnian/Famennian boundary interval in a fore-reef succession, Canning Basin, Western Australia. *Palaeogeography, Palaeoclimatology, Palaeoecology* **181**, 347-374 (2002). https://doi.org/https://doi.org/10.1016/S0031-0182(01)00485-0
35 Becker, R. T., House, M. R., Kirchgasser, W. T. & Playford, P. E. Sedimentary and faunal changes across the frasnian/famennian boundary in the canning basin of Western Australia. *Historical Biology* **5**, 183-196 (1991). https://doi.org/10.1080/10292389109380400
36 Klapper, G. FRASNIAN (UPPER DEVONIAN) CONODONT SUCCESSION AT HORSE SPRING AND CORRELATIVE SECTIONS, CANNING BASIN, WESTERN AUSTRALIA. *Journal of Paleontology* **81**, 513-537 (2007). https://doi.org/10.1666/05088.1
37 Morrow, J. R. & Sandberg, C. A. Evolution of Devonian carbonate-shelf margin, Nevada. *Affiliation (analytic): Moss Landing Marine Laboratories, Moss Landing, CA Affiliation (monographic): Moss Landing Marine Laboratories, Moss Landing, CA, United States Coordinates: N544012 N544012 W1695854 W1695854; N532354 N532354 W1793113 W1* **4**, 445-458 (2008). https://doi.org/10.1130/GES00134.1
38 Sandberg, C., Morrow, J. R., Poole, F. G. & Ziegler, W. Middle Devonian to Early Carboniferous event stratigraphy of Devils Gate and Northern Antelope Range sections, Nevada, U.S.A. *CFS Courier Forschungsinstitut Senckenberg*, 187-207 (2003).
39 Joachimski, M. M. & Buggisch, W. Conodont apatite δ18O signatures indicate climatic cooling as a trigger of the Late Devonian mass extinction. *Geology* **30**, 711-714 (2002). https://doi.org/10.1130/0091-7613(2002)030<0711:CAOSIC>2.0.CO;2
40 Johnson, J. G., Klapper, G. & Sandberg, C. A. Devonian eustatic fluctuations in Euramerica. *GSA Bulletin* **96**, 567-587 (1985). https://doi.org/10.1130/0016-7606(1985)96<567:DEFIE>2.0.CO;2
41 Ma, X. P. & Bai, S. L. Biological, depositional, microspherule, and geochemical records of the Frasnian/Famennian boundary beds, South China. *Palaeogeography, Palaeoclimatology, Palaeoecology* **181**, 325-346 (2002). https://doi.org/https://doi.org/10.1016/S0031-0182(01)00484-9
42 Huang, C. & Gong, Y. Timing and patterns of the Frasnian–Famennian event: Evidences from high-resolution conodont biostratigraphy and event stratigraphy at the Yangdi section, Guangxi, South China. *Palaeogeography, Palaeoclimatology, Palaeoecology* **448**, 317-338 (2016). https://doi.org/https://doi.org/10.1016/j.palaeo.2015.10.031
43 Chen, D. & Tucker, M. E. The Frasnian–Famennian mass extinction: insights from high-resolution sequence stratigraphy and cyclostratigraphy in South China. *Palaeogeography, Palaeoclimatology, Palaeoecology* **193**, 87-111 (2003). https://doi.org/https://doi.org/10.1016/S0031-0182(02)00716-2
44 Chen, D. *et al.* Large sulphur isotopic perturbations and oceanic changes during the Frasnian–Famennian transition of the Late Devonian. *Journal of the Geological Society* **170**, 465-476 (2013). https://doi.org/10.1144/jgs2012-037

45 Chen, X. *et al.* Diagenetic effects on uranium isotope fractionation in carbonate sediments from the Bahamas. *Geochimica et Cosmochimica Acta* **237**, 294-311 (2018). https://doi.org/https://doi.org/10.1016/j.gca.2018.06.026

46 Higgins, J. A. *et al.* Mineralogy, early marine diagenesis, and the chemistry of shallow-water carbonate sediments. *Geochimica et Cosmochimica Acta* **220**, 512-534 (2018). https://doi.org/https://doi.org/10.1016/j.gca.2017.09.046

47 Evans, D. *et al.* Trace and major element incorporation into amorphous calcium carbonate (ACC) precipitated from seawater. *Geochimica et Cosmochimica Acta* **290**, 293-311 (2020). https://doi.org/https://doi.org/10.1016/j.gca.2020.08.034

48 Dodd, M. S. *et al.* Development of carbonate-associated phosphate (CAP) as a proxy for reconstructing ancient ocean phosphate levels. *Geochimica et Cosmochimica Acta* **301**, 48-69 (2021). https://doi.org/10.1016/j.gca.2021.02.038

49 Ingalls, M. *et al.* P/Ca in Carbonates as a Proxy for Alkalinity and Phosphate Levels. *Geophysical Research Letters* **47**, e2020GL088804 (2020). https://doi.org/https://doi.org/10.1029/2020GL088804

50 Pogge von Strandmann, P. A. E. *et al.* Global climate stabilisation by chemical weathering during the Hirnantian glaciation. *Geochemical Perspectives Letters* **3**, 230-237 (2017). https://doi.org/10.7185/230 geochemlet.1726
